# Flexible porous microneedle array for bioelectric skin patch

**DOI:** 10.1007/s10544-025-00749-y

**Published:** 2025-05-10

**Authors:** Soichiro Tottori, Mirai Matsuura, Sae Ichinose, Haechang Cho, Tarryn Galloway, Natsuho Moriyama, Matsuhiko Nishizawa

**Affiliations:** 1 Department of Finemechanics, Graduate School of Engineering, Tohoku University, 6-6-1 Aramaki Aoba, Aoba-ku, Sendai, 980-8579 Japan; 2 Department of Biomedical Engineering, Graduate School of Biomedical Engineering, Tohoku University, 6-6-4 Aramaki Aoba, Aoba-ku, Sendai, 980-8579 Japan; 3 Department of Biomedical Engineering, Georgia Institute of Technology, Atlanta, GA 30332 USA; 4 bionto Co., Tohoku University, 468-1 Aramaki Aoba, Aoba-ku, Sendai, 980-8579 Japan

**Keywords:** Porous microneedle, Flexible device, Skin patch, Iontophoresis

## Abstract

Microneedles with porous internal structures can provide pathways for transdermal ionic current and drug delivery by penetrating the stratum corneum of the skin. However, conventional porous microneedle arrays are typically monolithic and rigid, limiting their flexibility and adaptability to curved skin surfaces. To address the issue, a method to directly integrate an array of porous microneedles to a flexible substrate is proposed, preserving their skin penetration capability while enhancing flexibility. The resulting array conforms to curved skin surfaces while effectively reducing transdermal ionic resistance. Numerical and analytical modeling demonstrates that the limited number of needles on a flexible array is sufficient to reduce transdermal resistance. Further, an enzymatic battery is combined to create a fully organic, porous microneedle-based bioelectric skin patch that can generate stable transdermal current suitable for stimulation and drug delivery applications.

**Supplementary Information:**

The online version contains supplementary material available at 10.1007/s10544-025-00749-y.

## Introduction

The development of effective, patient-friendly skin patches has garnered attention in recent years, particularly in the context of minimally invasive and self-administered treatments (Heikenfeld et al. 2019; Ray et al. 2019; Yang and Gao 2019). One such application is the electric skin patch, which utilizes direct current (DC) electricity for therapeutic effects, including iontophoretic drug delivery, wound healing, and health monitoring (Pikal 2001; Prausnitz 1996; Kai et al. 2017; Ogawa et al. 2015) while maintaining minimized invasiveness. With regard to the power source that drives the electric patches, for example, enzymatic biobatteries have attracted attention as a viable candidate due to their biocompatibility and soft structure, which align well with the requirements of wearable patch devices (Bandodkar and Wang 2016; Mano and de Poulpiquet 2017; Yoshida et al. 2020a). On the other hand, the stable passing of transdermal current could be hampered by the large DC resistance of stratum corneum, outermost layer of skin (~ MΩ·cm^2^) (Abe et al. 2021), especially in the use of low output power sources such as biobatteries. To address the issue, techniques such as ultrasound or microneedles have been explored to create conductive pathways through the skin (Prausnitz and Langer 2008).

Among various chemical and physical methods for reducing skin resistance, the use of microneedles (typically < 1 mm in length), which can penetrate the stratum corneum without reaching blood vessels or nerves, is appealing for its simplicity and minimal invasiveness (He et al. 2022; Prausnitz and Langer 2008; Zheng et al. 2024). In particular, solid porous microneedles (PMNs) with micro/nanochannel network throughout the needle structure offer a unique ionic pathway for iontophoretic drug delivery and collection of interstitial fluid (Bao et al. 2022; Kai et al. 2019; Kusama et al. 2021; Pang et al. 2024; Wang et al. 2024). Despite their potential, however, the rigid substrates of conventional PMNs are poorly suited for conforming to surfaces with high curvatures or areas subject to stretching, such as fingers, elbows, and faces (Huang et al. 2021); therefore, the development of flexible PMN arrays is essential for the skin conformability of the patch while reducing the transdermal DC resistivity. Previous efforts to fabricate flexible microneedles have focused mainly on the bonding of non-porous solid needles to elastomeric substrates (Gao et al. 2019; Lee et al. 2020, 2021; Li et al. 2019; Sadeqi et al. 2022; Vecchione et al. 2014; Woodhouse et al. 2021; Yin et al. 2018; Zhao et al. 2024). On the other hand, the flexible PMN arrays that has porosity through the substrate is inherently difficult to be fabricated and has not been investigated so far.

**Fig. 1:**
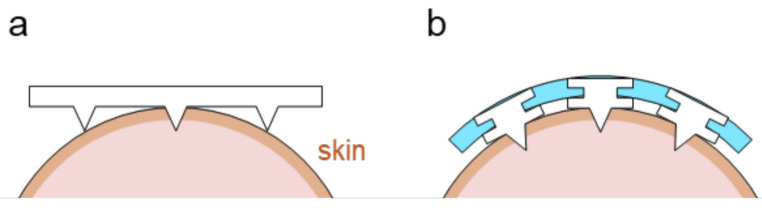
Array of porous microneedles formed on a rigid porous substrate (**a**) and that embedded in a flexible substrate (**b**)

In this study, we developed a PMN-mounted flexible patch using polydimethylsiloxane (PDMS) sheet for application on curved uneven skin surface (Fig. 1). The individual PMNs made of porous poly-glycidyl methacrylate were embedded into the PDMS sheets by mechanical interlocking technique (Yang et al. 2020). Since the back-end parts of each PMN are not covered with PDMS, it is possible to apply transdermal DC current through the PMN-based flexible patch. The application of the flexible patch reduced transdermal resistance by more than an order of magnitude, even on curved pig skin. The models of transdermal electric circuit using both finite element methods and analytical calculations showed that even with a limited number of needles, the patch contributes to significant reduction of resistance. Finally, a biobattery, consisting of enzyme-modified fabric electrodes, was integrated with the flexible PMN array to demonstrate stable transdermal current generation from glucose and oxygen, even when wrapped around a finger. The integration of the flexible PMNs array into bioelectric patches would advance the development of minimally invasive, totally organic therapeutic solutions.

## Materials and methods

### Fabrication of PMNs

The preparation of PMNs made of poly-glycidyl methacrylate (PGMA) was based on the previous protocol with minor modification (Liu et al. 2016; Wang et al. 2024). Briefly, a monomer stock solution was prepared by mixing glycidyl methacrylate (10 mL), trimethylolpropane trimethacrylate (5.23 mL) and triethylene glycol dimethacrylate (15.7 mL). A porogen stock solution was prepared by mixing polyethylene glycol (PEG, 10 kDa, 4.0 g) and diethylene glycol monomethyl ether (DEG, 20 g) at 50 °C. Immediately after mixing the monomer and porogen stock solutions at a volume ratio of 11:9, the photoinitiator Irgacure 184 was added at 1 wt%. This “PMN raw solution” was poured into the mold made of PDMS (SILPOT 184, Dow Inc.) and degassed under vacuum at − 0.096 MPa for 80 min. Photopolymerization was carried out by exposing the mixture to UV light (wavelength: 365 nm) for over 7 h at room temperature. The porogen (PEG) was dissolved by immersing in water-ethanol mixture (1:1 in volume) at 60 °C for 12 h. The porosity of PMNs was approximately 40% as estimated from the gravimetric water content (weight difference between the dry and water-swollen conditions) and was satisfactorily consistent with the theoretical value (ratio of porogen in the mixture) (Supplementary Figure S1), indicating that most of the porogen was dissolved through the interconnecting voids between the PGMA particles (Abe et al. 2022).

The individual PMN withstood an applied force of around 1.13 N, which is well larger than the load 0.098 N required for penetrating human skin (Wang et al. 2024).

The PDMS molds used in the above preparation protocol of PMNs was fabricated by two-step shape-transfer from the mother molds of poly (methyl methacrylate) machined with laser cutting system (Universal Laser System) and drilled by a 5-axis CNC machine (PRODIA M45, Modia Systems) with a square-end mill (diameter 300 μm, Nissin tool) and a conical drill bit (tip diameter < 10 μm, tip taper angle 13.5°, custom-made by Modia systems Co). The male PDMS mold was placed in a disposable cup and treated with plasma for 1 min, followed by the silanization of Trichloro(1 H,1 H,2 H,2 H-perfluorooctyl)silane for 6 h in a fume hood, followed by using for making the female PDMS molds.

### Fabrication of enzyme electrodes

The fabrication of enzyme biobatteries, consisting of bilirubin oxidase (BOD, BO-Amano-3 Amano Enzyme Inc.) for the cathode and glucose dehydrogenase (GDH, FADGDH-AD, Kikkoman Biochemifa Company) for the anode, is as follows, as previously described elsewhere (Terutsuki et al. 2022; Yoshida et al. 2020a).

#### BOD cathode electrode

A 40 mg sample of acid-treated CNTs (C 70 P; Baytubes, Bayer MaterialScience AG, Germany) and 0.2 mL of poly(3,4-ethylenedioxythiophene)-poly(styrenesulfonate) (PEDOT: PSS) (739 324-100G; Sigma-Aldrich Co., St. Louis, MO, USA) were dispersed using a homogenizer for 20 min under ice cooling. BOD was dissolved in a phosphate-buffered solution at a concentration of 200 mg·mL^–1^. The buffer solution was composed of 1.0 L of deionized water, 0.1 M of disodium hydrogen phosphate (197–02865; FUJIFILM Wako Pure Chemical Corporation, Ltd., Osaka, Japan) and 2 M of sodium hydroxide (198-13765; FUJIFILM Wako Pure Chemical Corporation, Ltd.). The resulting CNT dispersion and BOD solution were then mixed in a 1:1 ratio by pipetting. After applying 10 µL·cm^–2^ of ethanol to the piece of carbon fabric (CF) (Nakatsuyama Netsusyori Co., Ltd, Niigata, Japan), 50 µL·cm^–2^ of the BOD/CNT solution was applied and dried at − 0.1 MPa and 35 °C for 30 min. Finally, a 100 µL·cm^–2^ portion of the dispersion containing 4.0 mg·mL^–1^ CNTs and 12.5 mg·mL^–1^ polytetrafluoroethylene (PTFE) (165-13412; FUJIFILM Wako Pure Chemical Corporation, Ltd.) was applied to the BOD-modified CF for making its surface hydrophobic, followed by drying in an oven at 35^◦^C for 30 min. This process was then repeated twice.

#### GDH anode electrode

A 4.0 mg sample of CNTs, 0.8 mg of linker (1-pyrenebutanoic acid succinimidyl ester; PBSE) (sc-213409; Santa Cruz Biotechnology, Inc., TX, USA), 2.0 mg of mediator (9,10-phenanthrenequinone; 9,10-PQ) (P1136; Tokyo Chemical Industry Co., Ltd., Tokyo, Japan), and 0.4 mL of N, N-dimethylformamide were dispersed using a homogenizer. The dispersion was filtered, and the filter paper containing the CNTs was rinsed three times before being dried in a vacuum oven at − 0.1 MPa and 50 °C for 30 min. Subsequently, 50 µL of PEDOT: PSS and 3 mg of mediator- and linker-modified CNT powder were added to a potassium phosphate buffer (50 mM, pH 7), composed of a mixture of 26.8 mM KH_2_PO_4_ and 23.2 mM K_2_HPO_4_. After applying 10 µL·cm^–2^ of ethanol, 60 µL·cm^–2^ of CNT dispersion was applied to the 1 cm^2^ carbon fabric (CF), and the CF-CNT was dried in a vacuum at 70 °C for 10 min. Finally, 50 µL·cm^–2^ of GDH solution was drop-cast onto the CF-CNT and then dried at room temperature.

### Characterization of PMN-based flexible patches

The measurements of transdermal DC resistance were conducted using skin pieces (thickness: 4 mm) obtained from 6-month-old, non-pigmented, castrated male Landrace swine (DARD Corp., Tokyo, Japan). A PMN chip filled with phosphate-buffered saline (PBS) was placed and pressed on the pig skin at 5 mm/min with force monitoring with a digital force gauge (ZTA-5 N; IMADA Co., Ltd., Aichi, Japan). The resistance was calculated from the voltage generated on applying 1 µA between the commercial Ag/AgCl electrodes (NCS Electrode, NM-31, Nihon Kohden, Japan) placed on the upper and lower surface of the skin.

The measurement of transdermal current produced by the enzymatic battery was conducted using the pig skin pieces, enzyme-modified carbon fabric electrodes, the flexible PMNs array, pieces of polyurethane (PU) sponge with a thickness of 0.5 mm and dimensions of 12 mm by 12 mm (Soflus, AION Co., Ltd., Osaka, Japan) and O_2_-permeable polyurethane dressing (Cathereep, Nichiban, Japan). McIlvaine buffer (0.8 M Na_2_HPO_4_ + 0.4 M citric acid, pH 7.0) was absorbed into the PU sponges on the measurements.

## Results and discussion

### Fabrication of flexible PMN array

**Fig. 2:**
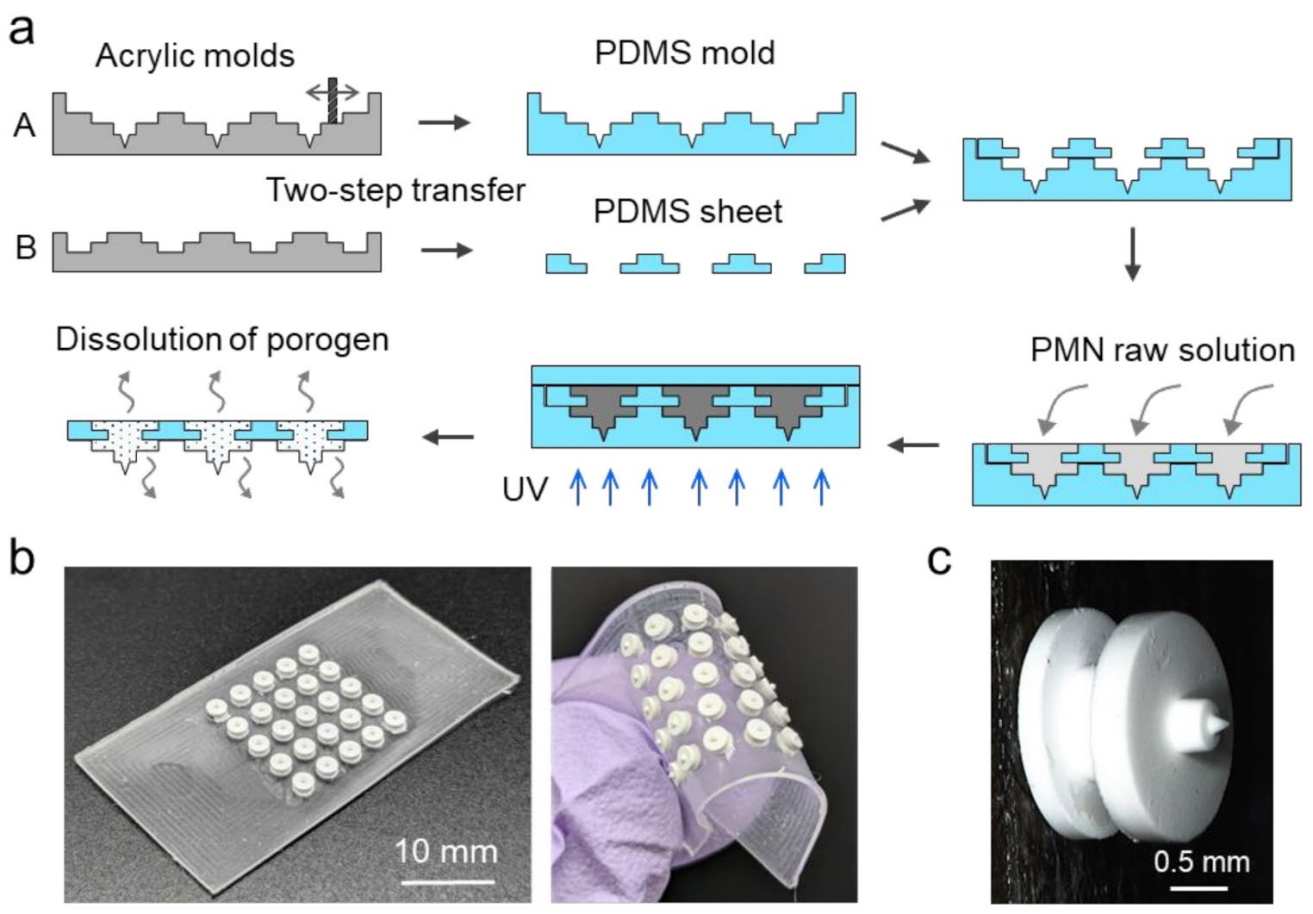
The flexible PMN array. (**a**) Procedure to embed PMNs within a flexible PDMS sheet by mechanical interlocking. (**b**) Photographs of the fabricated 5 × 5 PMNs array with flexible substrate. (**c**) Closed-up image of a PMN

The overall fabrication process of a flexible PMN array summarized in Fig. 2a is as follows (the blueprint of the PMN-embedded PDMS sheet is shown in Supplementary Figure S2): (1) Acrylic plates were milled using an endmill and a drill to prepare molds A and B. (2) The PDMS mold with the needle-shape cavities and the PDMS sheet with stepped holes (the substrate of PMN array eventually, 0.5 mm thick) were prepared through two-step shape-transfer from the acrylic molds A and B, respectively. (3) The PDMS mold and the PDMS sheet were aligned and laminated together. (4) The PMN raw solution, of which composition was described in the Methods section, was poured into the laminated construction and vacuumed to promote uniform penetration of the solution into the needle-shape cavities. (5) A PDMS block was placed to ensure back surface of the patch flat, followed by the polymerization of PGMA by exposing to UV light from the bottom side. (6) After removing from the PDMS mold, the patch was immersed in a water-ethanol mixture to completely dissolve the PEG porogen. Figure 2b shows the fabricated flexible PMN array with 5 × 5 needles at 3 mm intervals. It was confirmed that the PMNs do not detach from the flexible PDMS substrate during its bending and stretching by stable bonding through mechanical interlocking. The resulting flexible array conforms to curved surfaces with high curvature and can be wrapped around, for example, a gelatin rod with a diameter of 4 mm (curvature of 0.5 mm^− 1^) (Supplementary Figure S3). Figure 2c shows the geometry of the individual PMN to be composed of a cylindrical supporting post for reliable skin penetration (Nagamine et al. 2017).

### Characterization of flexible PMN array

**Fig. 3:**
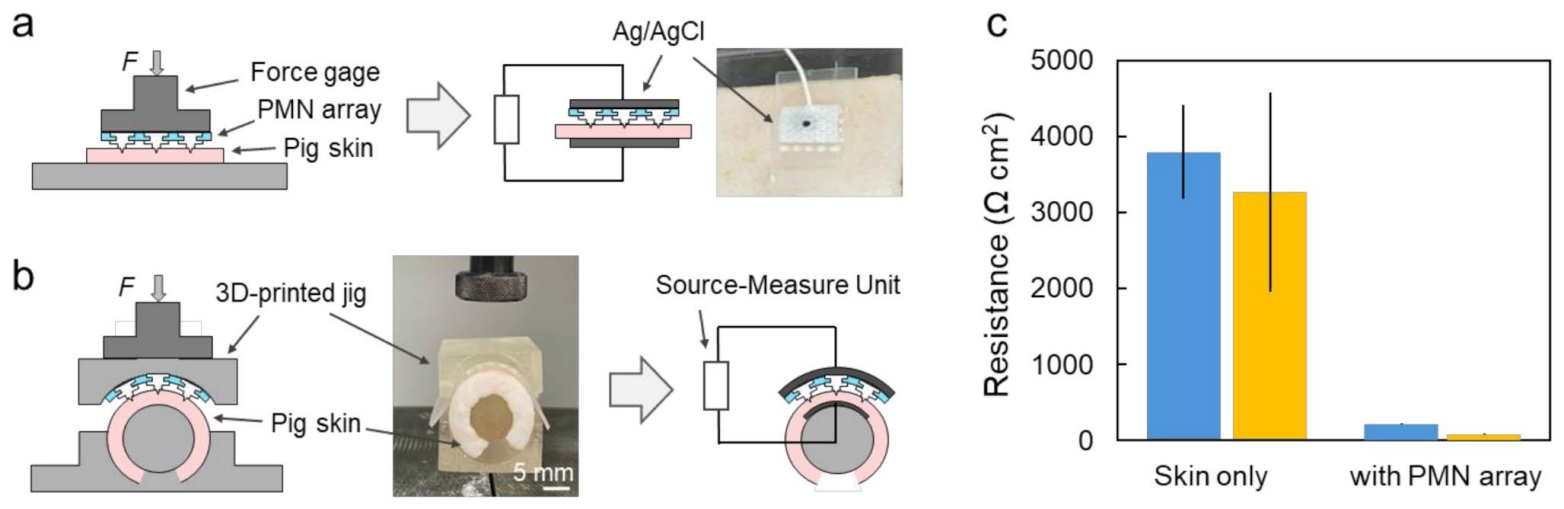
Electric resistance measurements of pig skins before and after the application of the flexible PMN array. (**a**, **b**) Experimental setup for penetration of the PMN array by uniform compress on the flat and curved surface of pig skins, respectively, followed by the resistance measurements. (**c**) DC resistance of flat and curved skin surfaces without and with the microneedle penetration. Error bars represent the standard errors (skin only: *N* = 10; with microneedle array: *N* = 3)

The DC resistance of the phosphate-buffered saline (PBS)-filled PMNs 5 × 5 array was measured on a 3wt% agarose gel prepared with PBS. The measured value was 0.63 ± 0.08 kΩ (*N* = 3), which corresponds to an approximate resistance of 15.8 kΩ per needle, examined negligibly low compared to that of of skin. Figure 3a and b show the setups for the measurements of transdermal DC resistances after loading of 30 N to the flat and curved pig skins, respectively. To prepare the curved condition, the skin pieces were wrapped around a plastic rod (9 mm in radius), and a 3D-printed curved jig was used to apply force evenly. After the force application, Ag/AgCl electrodes were placed on the upper and lower surface of the skin to measure transdermal DC resistance by a source-meter from the voltage drop upon the application of 1 µA. The resistance values were normalized by the effective contact area of the Ag/AgCl electrode, approximately 3.6 cm^2^. By applying the flexible PMN arrays, both the transdermal resistances of the flat and curved skins were significantly decreased from over 3000 kΩ·cm^2^ to below 250 kΩ·cm^2^ by more than an order of magnitude as shown in Fig. 3c. These results obtained demonstrate that the PMN array effectively penetrated the skin to form ionic pathways through the stratum corneum even on the curved skin. The resistance in the range of 1000 kΩ·cm^2^ and 100 kΩ·cm^2^ observed before and after the PMN applications are reasonable values for stratum corneum and epidermis layers (Abe et al. 2021). The slight difference in the resistance between the flat and curved cases is caused by internal conductivity changes due to deformation of electrodes or inherent variability in individual skin samples.

### Numerical simulation and analytical model of transdermal resistance

**Fig. 4:**
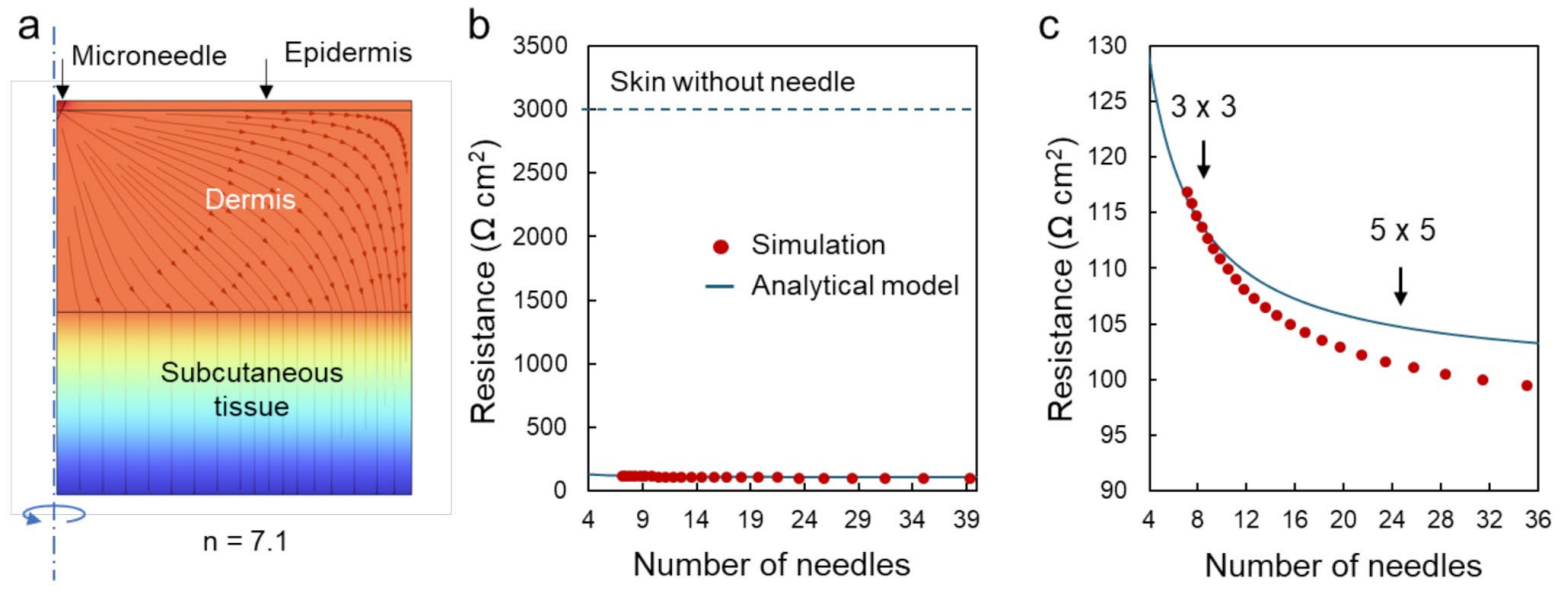
(**a**) An example of the simulated electric potential profile in the skin tissue around a microneedle. (**b**) The calculated transdermal resistance as a function of the number of needles. Solid lines and dots represent the analytical model and numerical simulation results, respectively. The typical resistance of skin without needle (3000 Ω cm^2^) is indicated by a dotted line. (**c**) The enlarged plot to show the detailed effect of number of needles on transdermal electrical resistance

The flexible PMN array developed here is limited in increasing the number and density of needles due to the physical bulkiness of the interlocking structure. To investigate the relationship between transdermal resistance and the number of needles after skin penetration, the numerical simulations using finite element method (COMSOL Multiphysics^®^ v. 6.2) was used to model electric potential profiles within skin tissue. The stratum corneum was omitted, as the model focuses on the scenario after the needles have penetrated the stratum corneum. The typical conductivities of each layer were set according to the previous reports (Abe et al. 2021; Khadka and Bikson 2020): epidermis, 1.0^5^ × 10^− 5^ S·m^− 1^; dermis, 2.3 × 10^− 1^ S·m^− 1^; subcutaneous tissue, 2.0 × 10^− 4^ S·m^− 1^; microneedle, 0.3 S·m^− 1^. The geometrical parameters were as follows: epidermis, 0.1 mm; dermis, 2 mm; subcutaneous tissue, 2 mm; microneedle height, 0.2 mm; and microneedle radius, 0.1 mm. The number of the needles, n, refers to the count per the total area of the bottom electrode (Ae = 3.6 cm^2^). Figure 4a shows an example of the simulated electric potential profile at *n* = 7.1 under the boundary condition detailed in Supplementary Figure S4. On the other hand, the analytical model of the transdermal resistance with the PMN array, $\:{R}_{t}$, is expressed as:

1 $$\:{R}_{t}=\frac{{R}_{n}+{R}_{a}}{N}+{R}_{d}+{R}_{s}$$

where $\:{R}_{n}$, $\:{R}_{a}$, $\:N$, $\:{R}_{d}$, and $\:{R}_{s}\:$ represent the resistance of a single PMN (Ω), the access resistance, the number of microneedles per square centimeter ($\:N=n/{A}_{e}$), the resistance of dermis (Ω·cm^2^), and the resistance of subcutaneous tissue (Ω·cm^2^), respectively. The access resistance is generally given by:

2 $$\:{R}_{a}=\frac{1}{4\sigma\:a}$$

where $\:\sigma\:$ is the conductivity of the media and $\:a$ is the radius of the entrance (Hall 1975). The entrance radius $\:a$ refers to the radius of a single needle, and the conductivity $\:\sigma\:$ correspond to that of the dermis. As shown in below, the analytical models align well with the numerical simulations, serving as a useful tool for rapidly estimating the resistance of skin with PMNs without requiring numerical simulation software.

Figure 4b summarizes the results of the simulation (dotted curve) and analytical model (solid curve) as a function of the number of needles, showing that the transdermal resistances are significantly decreased by the penetration of microneedle (*n* > 4) by more than an order of magnitude, in agreement with the experimental results in Fig. 3c. Importantly, this markedly significant reduction in transdermal resistance is roughly independent of the number of needles. Figure 4c is the enlarged plot to show the detailed effect of the number of needle. The transdermal resistance values gradually decreased and eventually plateau as the number of needles increases, approaching the resistance of the dermis and subcutaneous tissue. Furthermore, increases in the number of needles do not directly affect the decrease of resistance. For example, three times increase of needles from 9 (3 × 3) to 25 (5 × 5) causes just 15% decrease in the resistance. These results suggest that the relatively low needle density of the present PMN array due to mechanical interlocking structure does not diminish the needle’s resistance-reducing capability.

### Integration with enzymatic biobattery

**Fig. 5:**
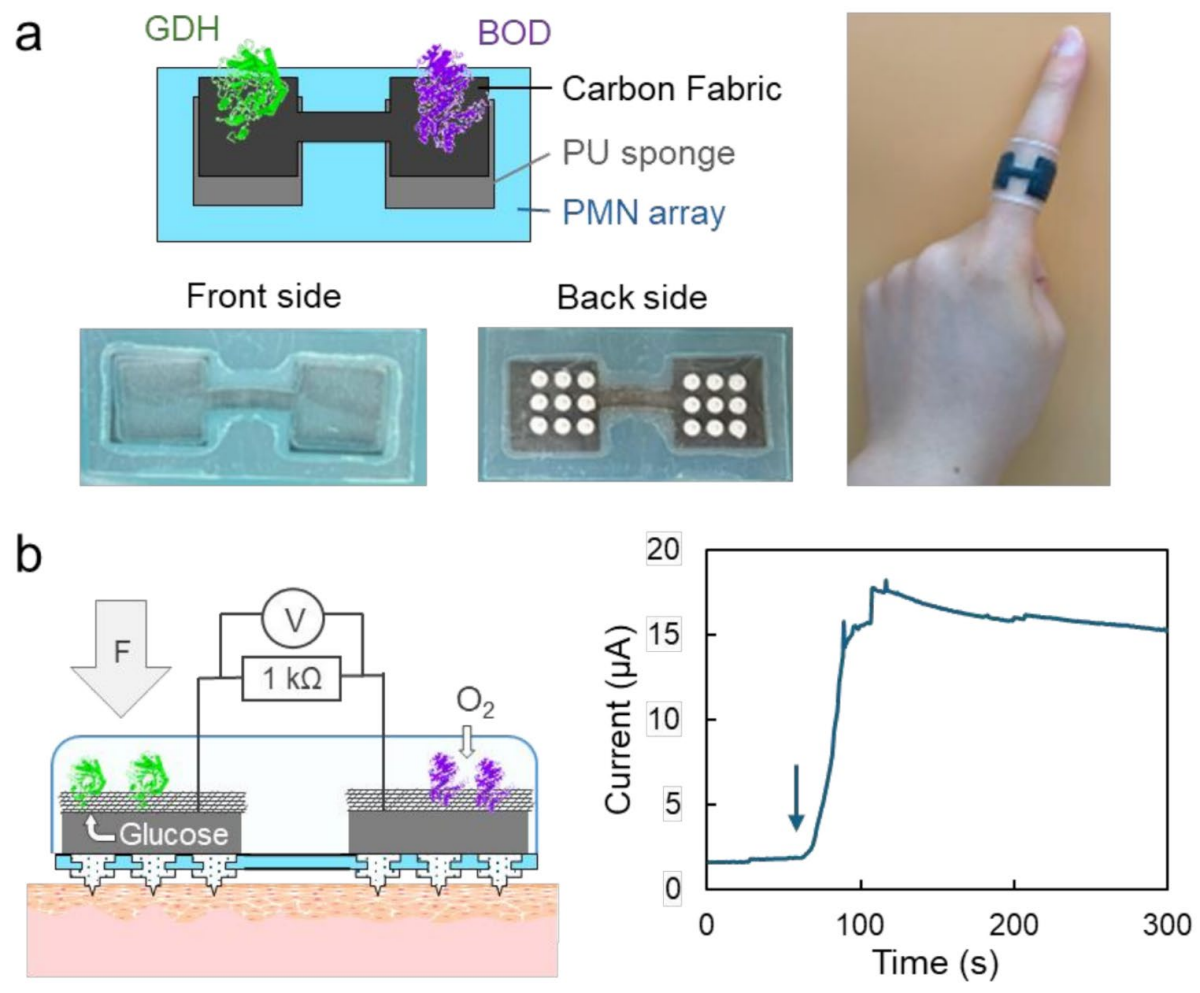
Integration with enzymatic biobattery. (**a**) Illustration and photographs of the flexible PMN array integrated with BOD-GDH enzymatic biobattery. (**b**) Transdermal current during the press of the patch onto a pig skin. The current value was monitored using 1 kΩ resister

Based on the validation of the effectiveness of flexible PMN arrays in reducing transdermal resistance, the integration of an enzymatic biobattery to promote microcurrents through the skin was studied. The enzymatic biobattery was prepared by a patterned carbon fabric modified with BOD and GDH for the cathode and the anode catalysts, respectively. The detailed preparation protocol is described in the Methods section. As shown in Fig. 5a, the BOD-GDH biobattery was sandwiched between the flexible PMN array and an O_2_-permeable PU dressing, enabling the wrapping around a finger.

As shown in Fig. 5b, the flexible PMN array (3 × 3), PU sponge and the BOD cathode and GDH anode were placed on top of each other on a pig skin piece. McIlvaine buffer with and without 200 mM glucose was absorbed into the PU sponges on the anodic and cathodic sides, respectively. Then, the transdermal current was monitored using a 1 kΩ resistor during the laminated configuration was gradually pressed at 5 mm/min using a force gauge up to a maximum force of 30 N. The transdermal current increased as soon as the force was applied and reached to the maximum of approximately 18 µA, more than 10 times promotion; the transdermal resistance was estimated to be roughly 50 kΩ from the separately obtained performance curve of the BOD/GDH battery (Supplementary Figure S5). The maximum current gradually decreased and reached a plateau, most likely due to the permanent skin deformation. As reported previously (Yoshida et al. 2020b; Kusama et al. 2021), the output current and voltage can be further increased by serially connecting multiple anodes and cathodes of the biobattery. Furthermore, the use of the specially designed ultrathin PDMS film (Terutsuki et al. 2022) instead of the PU dressing used here will provide the waterproof property expanding the use cases of the flexible bioelectric patch even in water and wet environment.

## Conclusion

A flexible PMN array has been developed by embedding PMNs within a flexible PDMS substrate by mechanical interlocking. Supported by the experiments using the pig skin pieces, transdermal resistance is demonstrated to be significantly reduced by more than an order of magnitude even on curved skin. The models of transdermal electric circuit using both finite element methods and analytical calculations shows that even a limited number of needles significantly reduce the resistance. The enzymatic biobattery delivered stable microcurrents through the developed flexible PMN array even when wrapped around a finger. The flexible PMNs array-based bioelectric patches would advance the development of minimally invasive, totally organic therapeutic solutions. Future work may explore the use of materials other than PDMS, such as flexible porous materials, to further enhance flexibility and improve drug or fuel loading capacity.

## Electronic supplementary material

Below is the link to the electronic supplementary material.

Supplementary Material 1

## Data Availability

Data sets generated during the current study are available from the corresponding author on reasonable request.
