# Supplementary material for: Flexible porous microneedle array for bioelectric skin patch
Source: Biomed Microdevices. 2025 May 10;27(2):21. doi: 10.1007/s10544-025-00749-y (PMC12065680; doi:10.1007/s10544-025-00749-y)
Supplement: Supplementary file 1 — Supplementary Material 1 [file 10544_2025_749_MOESM1_ESM.pdf]

Supplementary Information

Flexible Porous Microneedle Array for Bioelectric Skin Patch

Soichiro Tottori, Mirai Matsuura, Sae Ichinose, Haechang Cho, Tarryn Galloway, Natsuho Moriyama, Matsuhiko Nishizawa

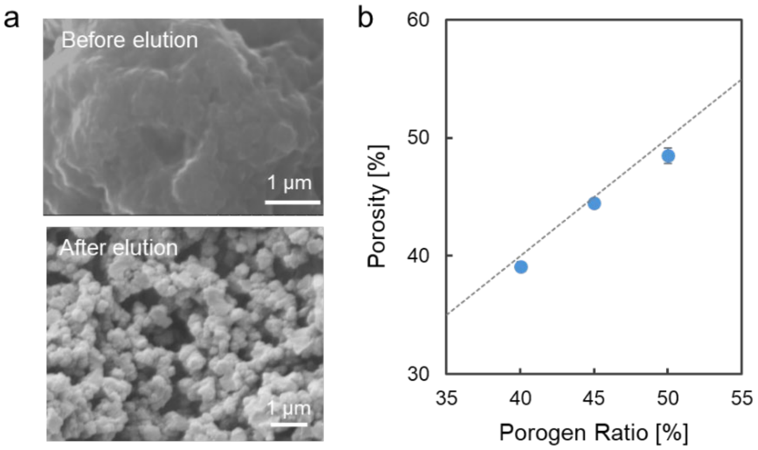

Figure S1 (a) SEM images of the microneedle before and after elution of PEG porogen.
(b) The theory (dotted line) and the measured porosities for different porogen ratio PMNs (n=3).

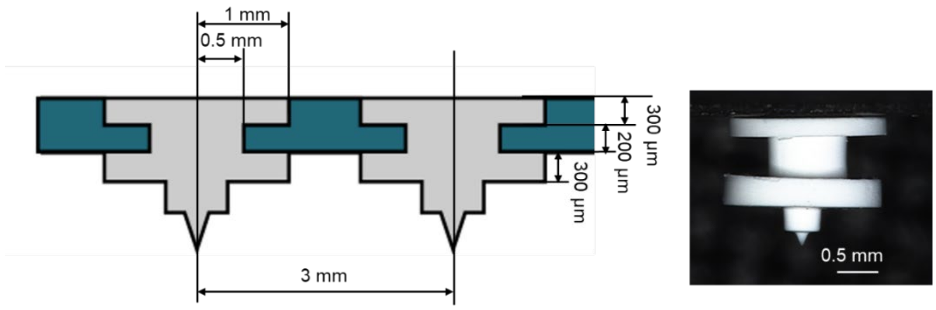

Figure S2 The blueprint of the PMN-embedded PDMS sheet and a photograph of a PMN.

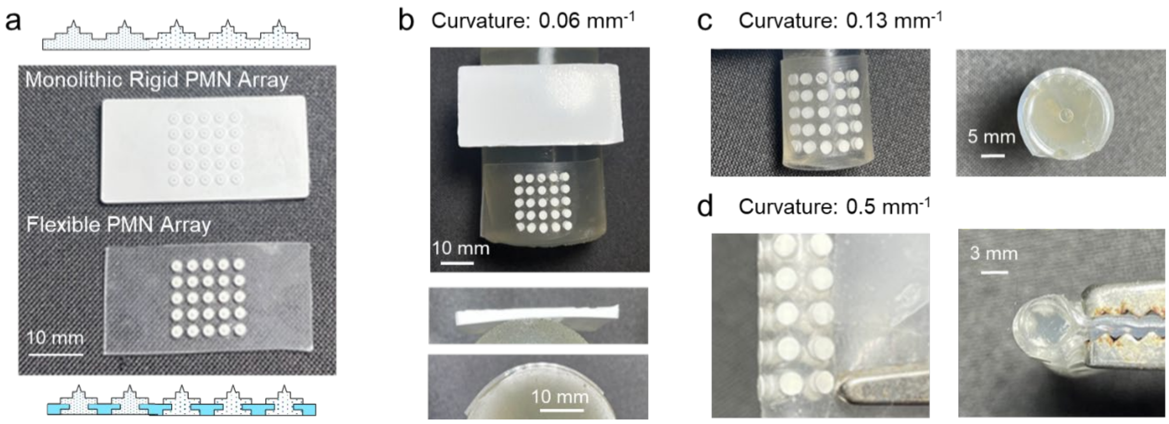

Figure S3 (a) Photographs of a monolithic rigid PMN array and a flexible PMN array of 5 x 5 needles with 3 mm intervals. For the monolithic array, also the substrate was made of porous PGMA. (b) The top and sectional views of the rigid and flexible arrays on a 10 wt% gelatin rod with a diameter of 36 mm (curvature of 0.06 mm^{-1}). (b, c) The flexible array wrapped to the rods with diameter of 15 mm and 4 mm (curvature of 0.13 mm^{-1} and 0.5 mm^{-1}).

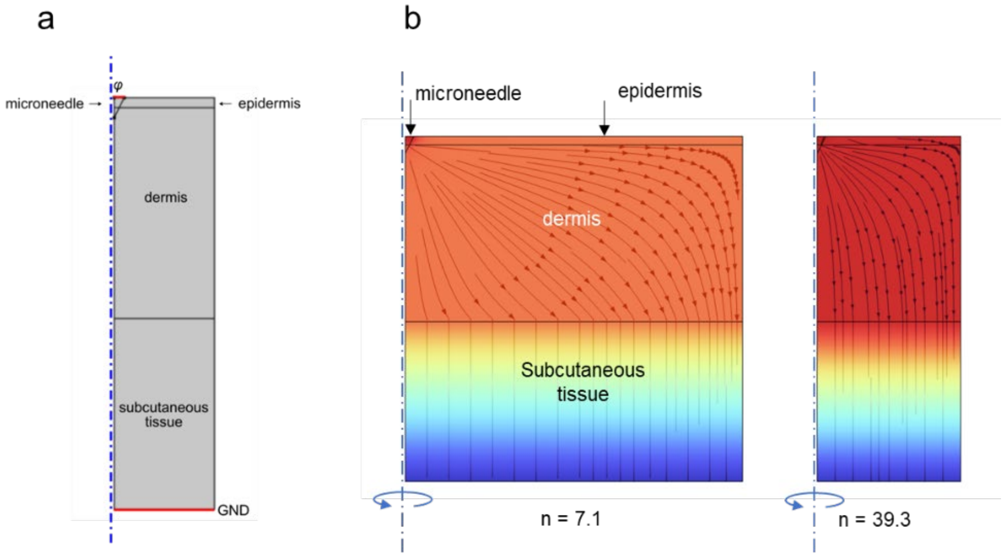

Figure S4 (a) Boundary conditions for numerical simulations of 2D axisymmetric resistance model. (b) Color maps of the electric potential profile in the needle and the surrounding skin tissue. With a relatively small number of needles (n = 7.1), the applied potential drops near the needle, indicating a reduction in the current density through the skin. As the number of needles increases (n = 39.3), the location of the potential drops gradually shifts deeper into the skin tissue. This occurs because the resistance near the needle becomes smaller relative to that of the dermis and subcutaneous tissue, resulting in a higher current density through the skin.

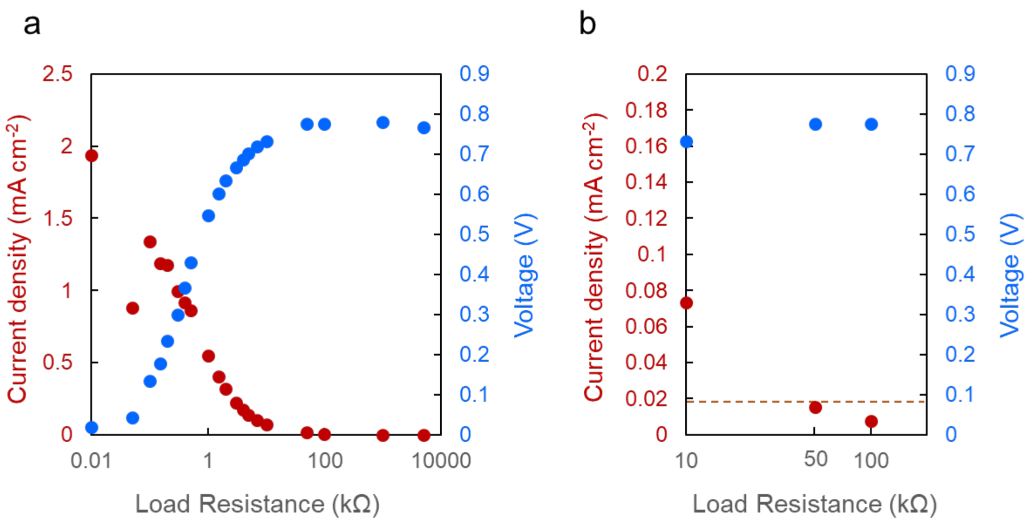

Figure S5 (a) The performances of a GDH / BOD battery measured by varying the load resistance (10 Ω ~ 5 MΩ) on a cotton wetted with McIlvaine buffer with 200 mM glucose. (b) The enlarged plot to show the detailed relationship between the resistance and the current values.
